# Subclinical Atrial Fibrillation on Prolonged ECG Holter Monitoring: Results from the Multicenter Real-World SAFARI (Silent Atrial Fibrillation ANCE-Sicily Research Initiative) Study

**DOI:** 10.3390/jcdd10080336

**Published:** 2023-08-04

**Authors:** Cesare de Gregorio, Antonino Di Franco, Antonio Vittorio Panno, Marco Di Franco, Giuseppe Scaccianoce, Francesca Campanella, Giuseppina Novo, Alfredo Ruggero Galassi, Salvatore Novo, the SAFARI Study Group

**Affiliations:** 1 Department of Clinical and Experimental Medicine, Cardiology Unit, University Hospital of Messina, 98122 Messina, Italy; 2 Department of Cardiothoracic Surgery, Weill Cornell Medicine, New York, NY 10065, USA; antoninodifranco.md@gmail.com; 3 Outpatient Cardiology Office, 90100 Palermo, Italy; 4 Outpatient Cardiology Office, 95100 Catania, Italy; 5 Department of Health Promotion, Mother and Child Care, Internal Medicine and Medical Specialties, Division of Cardiology, University of Palermo, 90133 Palermo, Italy; giuseppina.novo@unipa.it (G.N.); alfredo.galassi@unipa.it (A.R.G.); salvatore.novo@unipa.it (S.N.)

**Keywords:** arrhythmias, atrial fibrillation, chronic heart disease, electrocardiography, Holter monitoring devices

## Abstract

Background: The detection of subclinical/silent atrial fibrillation (SAF) in the general population is of the utmost importance, given its potential adverse consequences. Incident AF has been observed in 30% to 70% of patients with implanted devices, but its prevalence may indeed be lower in the general population. The prospective, multicentric, observational Silent Atrial Fibrillation ANCE Research Initiative (SAFARI) study aimed at assessing the SAF prevalence in a real-world outpatient setting by the means of a small, wearable, prolonged ECG Holter monitoring (>5 days) device (CGM HI 3-Lead ECG; CGM TELEMEDICINE, Piacenza, Italy). Methods: Patients ≥ 55 years of age at risk for AF were screened according to the inclusion criteria to undergo prolonged 3-lead ECG Holter monitoring. SAF episodes were classified as follows: Class A, <30 s; Class B, 30 to 299 s; and Class C, ≥300 s. Results: In total, 119 patients were enrolled (64 men; median age 71 (IQR 55–85) years). At a median of 13.5 (IQR 5–21) days of monitoring, SAF episodes were found in 19 patients (16%). A total of 10,552 arrhythmic episodes were registered, 6901 in Class A (n = 7 patients), 2927 in Class B (n = 3), and 724 in Class C (n = 9), (Class A vs. B and C, *p* < 0.001). This latter group had multiple (all-class) episodes, and two patients had >1000 episodes. There were no clinical, echocardiographic, or laboratory findings able to discriminate patients with SAF from those in sinus rhythm in univariate and multivariable analyses; of note is that the Class C patients showed a higher diastolic blood pressure, resting heart rate, and indexed LA volume. Conclusions. Over a median of 13 days of Holter monitoring, the SAFARI study confirmed the usefulness of small wearable devices in detecting SAF episodes in real-world outpatients at risk for, but with no prior history of, AF.

## 1. Introduction

Atrial fibrillation (AF) is associated with an increased risk of stroke and vascular dementia, especially in the elderly. The identification of AF in the general population is paramount, given its potential sequelae in terms of death and stroke occurrence [1,2,3,4].

Early studies have demonstrated that paroxysmal AF can occur even in the absence of symptoms and/or specific predictors, because of the unpredictability of situational/emotional stressors, physical activity, and other triggers, especially in subjects over 60 years of age [2,3,4,5]. Moreover, it has been demonstrated that even short-term atrial high-rate episodes (AHREs) detected by implanted atrial catheters could increase thromboembolic risk, like subclinical/silent AF (SAF) [3,4,5,6,7,8].

Incident AF has been estimated to occur in 30 to 70% of patients with permanent device monitoring, who are presumably affected by advanced heart diseases. However, its prevalence might be lower in the general population [2,3,7,8,9,10,11].

Current guidelines reinforce the need for detecting SAF, especially in those settings at a higher risk of cryptogenic stroke and in patients aged >55 years [3,8]. Various cost-effective strategies have been proposed for this purpose, such as opportunistic screening or systematic population screening. Miniaturized loop recorders, Bluetooth smartphones, wristwatches, or record-bar on demand recording, and small ECG monitoring devices have been demonstrated to be useful in detecting paroxysmal arrhythmias such as SAF. The use of extended external ECG monitoring is encouraged when the data from standard 24 h monitoring or implantable loop-recorders are inconclusive. Several ECG monitoring devices are available, although most are limited to 72 h recording [11,12,13,14,15]. Of note is that several scores (including machine-learning-based probability scores, surface-ECG-based scores, and artificial intelligence algorithms) have recently been tested and achieved a good predictive performance for AF recurrence in a range of different settings, allowing for tailored therapeutic approaches for individual patients [16,17,18].

The Silent Atrial Fibrillation ANCE-Sicily Research Initiative (SAFARI) study aimed at investigating the SAF prevalence in a real-world, clinically stable, cardiovascular patient population, by the means of prolonged external ECG Holter monitoring (>5 days).

## 2. Materials and Methods

### 2.1. Study Design

The SAFARI was a prospective, multicentric, observational study enrolling outpatients referred for stable cardiovascular disease, either as a de novo visit or follow-up. This study was conceptualized by the Sicilian Section of the Nationwide Scientific Society ANCE (*Cardiologia Italiana del Territorio*) Society (https://www.ancecardio.it (accessed on 3 August 2023).

Adult participants ≥ 55 years of age were enrolled on a voluntary basis if they had at least one of the pre-specified inclusion criteria, which are listed in Supplementary Table S1. Furthermore, the participants were required to have at least one of the following features: (a) supraventricular premature beats on a standard ECG; (b) palpitations of unknown origin; (c) a previous transient ischemic attack (TIA) or minor stroke; (d) evidence of gliosis of unknown origin on a brain magnetic resonance imaging/computed tomography (MRI/TC) scan; and (e) right or left atrial (LA) enlargement at echocardiography (the full list of inclusion criteria is reported in Supplementary Table S1). Any other unlisted condition was discussed by the steering committee of the study on a case-by-case basis. Patients with advanced cardiovascular diseases, severe heart failure, a prior pacemaker or defibrillator implantation, cancer, or severe kidney, liver, or lung disease were excluded.

The screening of eligible patients and ECG Holter recordings took place from April to December 2022. The flowchart outlining the study design is displayed in Supplementary Figure S1. Only data from ECG monitoring lasting at least 5 days were included in the analysis. Upon enrolment, a thorough clinical evaluation was conducted, inclusive of cardiovascular medical history and a comprehensive assessment of risk factors and major comorbidities. Standard 12-lead ECG, transthoracic 2D color-Doppler echocardiography and blood sample analyses were performed for all patients.

### 2.2. Diagnosis of Arrhythmias and ECG Monitoring Device

SAF episodes were defined as the occurrence of irregular R-R intervals in the absence of detectable P-waves and classified as follows:

- Class A: SAF lasting <30 s (at least 5 repetitive supraventricular beats);
- Class B: SAF episodes lasting from 30 to 299 s;
- Class C: SAF episodes lasting ≥300 s.

Each researcher was given 2 devices, model CGM HI 3 LEADS ECG devices (manufactured by CGM TELEMEDICINE Srl, Piacenza, Italy). Each one was associated with a smartphone (LG-X430EMW; LG Group, Seoul, Republic of Korea) equipped with the CGM CARE MAP mobile app. Through a Bluetooth connection, the ECG signal was unceasingly conveyed to the smartphone and then to the WEB platform CGM CARE MAP, medical device class IIA (installed on a CGM TELEMEDICINE server farm ISO 27001 certified for data protection, cloud service, and cloud computing). ECG files were available for online visualization and analysis by each researcher and the Central Core Lab (Supplementary Material) using dedicated analysis tools integrated into the CGM CARE MAP platform. A daily report was sent to the cardiologist for clinical use and overview. An arrhythmic alert was promptly conveyed to the clinician upon diagnosis, and her/his therapeutic management was unconditioned. ECG monitoring <5 days in length was not analyzed.

### 2.3. Training on Device and Software Use

Before starting the patient enrolment, each researcher attended a 4 h training session by two expert technicians of the CGM TELEMEDICINE industry. The live session was aimed at optimizing the management of the device, mobile phone, facilities, and tools, as well as the online software platform to register the patient data, analyze and elaborate the ECG file records.

### 2.4. Echocardiography

Echocardiography was performed using different machines equipped with 1.5–3.75 MHz transducers, with a thorough assessment of conventional measurements, chamber size, systolic and diastolic function, and valvular flow, according to current recommendations [19].

### 2.5. Clinical Assessment

Resting or exertional palpitations, dyspnea, and/or chest pain were assessed during a medical history interview, along with exercise tolerance, daily life limitations, and New York Heart Association (NYHA) class.

### 2.6. Ethical Considerations

This study was conducted in accordance with the principles stated in the Declaration of Helsinki. Written informed consent was obtained from all the participants at the time of performing the device testing. Ethical approval was granted on 15 February 2022 by the Institutional Review Board of the Paolo Giaccone University Hospital of Palermo, Palermo (Italy) (#02/2022). Members of the Steering Committee and the full list of the ANCE SAFARI Study group investigators are reported in Appendix A.

### 2.7. Statistical Analysis

The data are reported as median and inter-quartile range (IQR) or mean ± standard deviation as appropriate for continuous variables, and number and percentage for categorical variables. The Mann–Whitney U test, ANOVA analysis of variance, Bonferroni or chi-square, and Fisher’s exact test were used. We also applied linear regression and binary logistic Wald stepwise backward regression to examine the association between the clinical, laboratory, or echocardiographic findings and the occurrence of AF. The null hypothesis was rejected at two tails for *p* < 0.05. The analyses were performed using the IBM SPSS Statistics software, version 25.

## 3. Results

### 3.1. Patient Population and Arrhythmic Events

**Figure 1:**
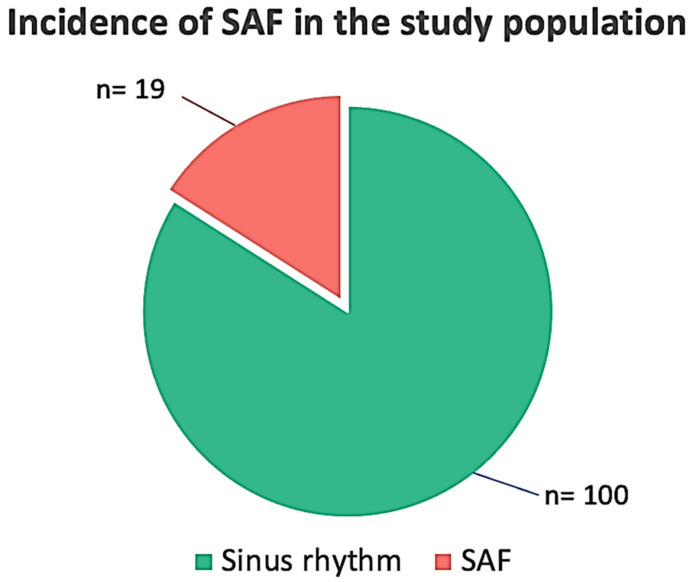
Incidence of silent/subclinical atrial fibrillation (SAF) in the study population.

A total of 145 patients were screened. In total, 10 patients were excluded because of severe mitral or aortic valve disease (n = 3), severely depressed left ventricular (LV) function (LVEF < 35%) (n = 4), and permanent AF or a previous history of paroxysmal AF (n = 3). After enrolment, 16 patients from the remaining 135 were excluded due to missing laboratory data or ECG monitoring lasting <5 days, due to patch intolerance in spring and summertime. The final study population consisted of 119 patients (64 men), with a median age of 71 (IQR 55–85) years, whose demographic and clinical characteristics are reported in Table 1. SAF episodes were found in 19 patients (16%) (Figure 1).

**Figure 2:**
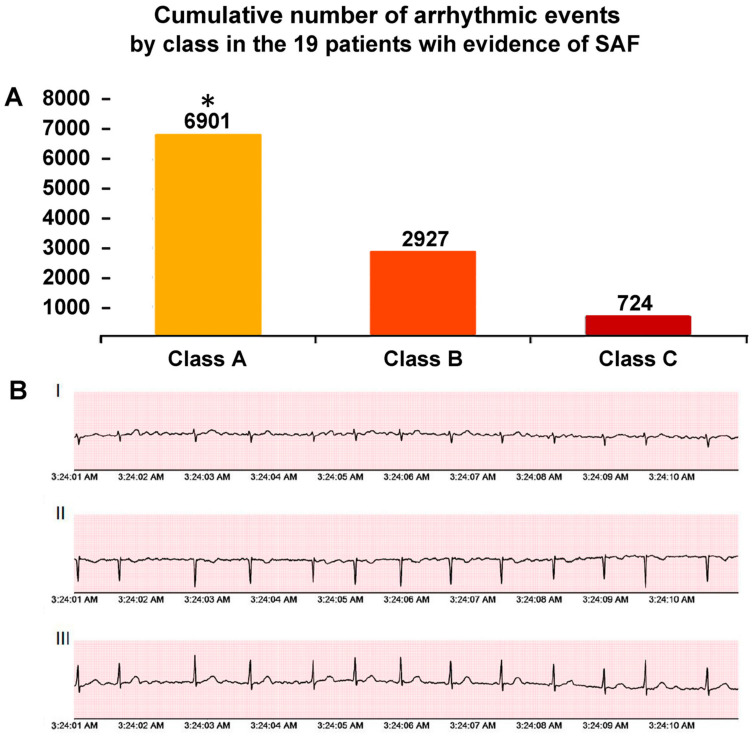
(Panel **A**). Cumulative number of arrhythmic events by class in the 19 patients with evidence of silent/subclinical atrial fibrillation (SAF). Class A SAF: <30 s; Class B SAF: 3′-299 s; and Class C SAF: ≥300 s. (Panel **B**). Representative episodes of SAF at three-lead Holter monitoring. * *p* < 0.001 (Class A vs. B and C). Abbreviations: I = lead D1; II = lead D2; III = lead D3.

The median length of the ECG Holter monitoring in the study population was 13.5 (IQR 5–21) days. A total of 10,552 arrhythmic episodes were recorded. As expected, Class A episodes (n = 6901) occurred more frequently than Class B (n = 2927) and C (n = 724) episodes (Figure 2). Nine out of the nineteen patients with arrhythmic events (47.4%; 7.6% of the total study population) had episodes belonging to different arrhythmic classes. Two patients showed very frequent events (>1000) during 12-day and 14-day monitoring, respectively. An episode of non-sustained ventricular tachycardia was detected in one patient with ischemic heart disease. No atrio-ventricular blocks were observed.

There were no clinical, echocardiographic, or laboratory findings able to discriminate patients with SAF from those in sinus rhythm (SR) in the univariate and multivariable stepwise backward regression analyses. Of note is that a history of atypical chest pain, palpitations, and dyspnea were similar between the groups. Beta blocker use was more frequent in the SAF group (Table 1).

### 3.2. Sub-Analysis of the Arrhythmic Group

The main clinical and echocardiographic characteristics of the SAF group are displayed in Table 2. Compared to Class A and B, the Class C patients were slightly older and had a lower systolic blood pressure and higher diastolic blood pressure and resting heart rate. The indexed LA volume was also significantly larger in this group.

### 3.3. Device’s Technical Strengths and Weaknesses

The CGM HI 3-Lead ECG device is a small and easy-to-wear tool, with good-quality three-channel ECG recordings. As for similar devices, interferences and instability of the ECG trace occurred in the case of sweat and moisture on the electrodes or in the case of incidental patch detachment. In late spring and summertime, most patients (mainly women) complained of patch-related skin irritation and redness after three days of monitoring.

This problem needed to be addressed upon electrode patch removal or new device application, so the decision was made by the Steering Committee to pause the study from 30 June to 10 September 2022. Enrolment was restarted thereafter. An important weakness of the device was a cracking tendency of the central rubber element devoted to covering the electrical connections, mainly occurring upon removal by the patient, such as when taking a shower or at the end of the monitoring. This problem often implied an interruption of the ECG trace recording and needed a replacement of the device.

## 4. Discussion

The SAFARI study investigated the incidence of SAF in a real-world setting of outpatients with chronic cardiovascular disease. A total of 10,552 atrial arrhythmic episodes were recorded in 19/119 patients (16%), distributed across three classes according to the length of the episodes. No clinical, laboratory, or echocardiographic predictors were identified in the multivariable analyses. Sustained SAF episodes (Class C) were observed in nine patients (7.6%); notably, these subjects had a larger indexed LA volume, as well as a higher heart rate at baseline, as compared to those from Classes A and B.

Of note is that a not trivial proportion of the SAF patients complained of palpitations and atypical chest pain, so that the “subclinical” definition may not strictly be accurate.

Although previous studies on incident AF have been performed with similar ECG-monitoring devices, the characteristics of patients, study design, protocols, and use of device, as well as the study timeline, were quite different, and this may hinder the generalizability of the results [12,13,14,15,20]. Nevertheless, the use of easy-to-wear small devices has been validated in various studies. Sanna analyzed the main characteristics of several devices, including standard ECGs, snapshot single-lead recordings with professional or personal devices (e.g., smartphone), Holter monitors, external tools with long-term recording capabilities, mobile cardiac outpatient telemetry, and cardiac implantable electronic devices. While most of these systems are effective in detecting AF, their sensitivity, specificity, and patient tolerability varied significantly. In addition, the degree of patient cooperation and compliance is an important predictor of successful long-term recordings [21].

In 2019, Wasserlauf et al. examined 31,349 h of smart-watch recordings in 24 ambulatory patients and showed a high sensitivity for AF detection and assessment of AF duration, with a positive predictive value of 39.9% and optimal patient tolerance [22]. Patient-activated devices have also been demonstrated as effective in highly selected patients with paroxysmal palpitations suggestive of subclinical arrhythmias [20].

Our results are consistent with a recent study testing the myBeat device on 150 patients, in which, at a median time of 4.95 ± 0.03 days, newly detected AF episodes occurred in 7.7% of the patients, and the rate of AF detection was significantly higher in the myBeat group (10.7%) than in the controls (4.7%) [23].

As expected, in arrhythmic disease settings, the occurrence of SAF and related thromboembolic risk is greater than that in the general population. In 430 patients with hypertrophic cardiomyopathy (HCM), Yashiro et al. showed that AF was an independent determinant of HCM-related death (adjusted hazard ratio 3.57, *p* < 0.001) and sudden death (adjusted hazard ratio 2.61, *p* = 0.038). As the patients with AF were divided into subgroups with paroxysmal AF or non-paroxysmal AF, only paroxysmal AF was identified as an independent prognostic factor, irrespective of whether the AF was subclinical or not [24].

The chance detection of paroxysmal AF increases with the length of monitoring system. Recently, 115 post-ablation patients (median age of 64 years) were investigated using AliveCor Kardia ECG recording and Holter monitoring. The two systems detected AF recurrences in 25.2% and 14.8% of the patients, respectively (*p* < 0.001), and the authors pointed out the fact that 2 weeks of ECG monitoring was the most useful timeline for the detection of AF recurrences in outpatient populations [25]. In line with this concept, the median duration of the ECG monitoring in our group was 13 days.

When thinking of prolonged external ECG Holter monitoring, the major concern is the use of patch electrodes, due to the risk of skin redness and allergy, especially in summertime. The technological miniaturization of electrodes will surely facilitate a wider use of such devices in patients at risk for stroke, without additional economic impact on the healthcare system [26].

### 4.1. Clinical Implications

Guidelines recommend anticoagulation for most patients with AF to prevent systemic thromboembolism, irrespective of whether their episodes are paroxysmal, persistent, or permanent [3,8]. The probability of detecting very brief episodes of SAF with a standard ECG recording is extremely low but increases with the use of Holter monitors and implanted devices [3,8,11,12,25,26,27]. However, clinical outcomes related to atrial arrhythmias in more complex patients may not apply to the general population or in patients with neurogenic (“lone”) AF [3,8,9,10].

The present study showed SAF occurrence in 16% of patients; however, only 7.6% of these events were longer than 5 min. While a wide range of SAF duration cut-offs (even a few seconds) have been reported to be associated with thromboembolism [28,29], episodes longer than 5 min were considered at a higher risk for thromboembolism [3]. It remains to be better elucidated whether shorter episodes should also be considered at a high risk in patients >55 years of age.

### 4.2. Study Limitations

Several limitations of this study should be acknowledged. The relatively small sample size limits the generalizability of the present results. As brain MRIs or CT scans were not available for all the patients, we were unable to perform a stroke risk analysis. Echocardiographic findings were available for 85% of the patients.

Although overall reliable alerts were given when arrhythmic events occurred, false positive and negative episodes might occur in patients with low-voltage p waves or muscle interferences. Studies with larger cohorts and longitudinal follow-ups are ongoing and expected to shed further light on this topic [30].

## 5. Conclusions

Over a median of 13 days of ECG Holter monitoring, SAF episodes were detected in 16% of our study population, with a duration of >300 s in 7.6% of cases. The SAFARI study confirmed the usefulness of small wearable devices in detecting subclinical arrhythmias in real-world outpatients at risk for, but with no prior history of AF. The prognostic impact of SAF episodes still needs further clarification.

## Figures and Tables

**Table 1:** Characteristics of study population and between-group differences.

	All (n = 119)	SAF (n = 19)	SR (n = 100)	*p*-Value
Age (years)	71 ± 9	70.9 ± 8.9	71.2 ± 10.2	0.440
Males	64 (54%)	11 (58%)	53 (53%)	0.446
Females	55 (46%)	8 (42%)	47 (47%)	0.450
Systolic blood pressure, mm Hg	123 ± 22	128 ± 25	122 ± 22	0.885
Diastolic blood pressure, mm Hg	87 ± 21	89 ± 18	88 ± 22	0.272
Resting heart rate, bpm	69 ± 8	67 ± 7	69 ± 8	0.886
BSA, m^2^	1.85 ± 0.19	1.92 ± 0.17	1.83 ± 0.19	0.126
NYHA Class	1.9 ± 0.5	2.0 ± 0.4	1.8 ± 0.7	0.409
Obesity	50 (42%)	10 (53%)	40 (40%)	0.259
Hypertension	88 (74%)	16 (84%)	72 (72%)	0.409
Diabetes	18 (15%)	4 (21%)	14 (14%)	0.348
Dyslipidemia	77 (65%)	12 (63%)	65 (65%)	0.493
Chronic ischemic heart disease	11 (9%)	3 (16%)	8 (8%)	0.245
Previous coronary stenting	7 (6%)	3 (16%)	4 (4%)	0.083
History of palpitations	94 (79%)	15 (90%)	79 (79%)	0.431
History of dyspnoea	36 (30%)	7 (37%)	29 (29%)	0.685
History of atypical chest pain	18 (15%)	3 (16%)	15 (15%)	0.650
Hypertrophic cardiomyopathy	3 (2.5%)	1 (5%)	2 (2%)	0.591
Peripheral artery disease	13 (11%)	4 (21%)	9 (9%)	0.129
Cerebro-vascular disease/gliosis	19 (16%)	4 (21%)	15 (15%)	0.357
Chronic obstructive pulmonary disease	13 (11%)	0 (0%)	13 (13%)	0.091
OSAS	9 (8%)	2 (10%)	7 (7%)	0.438
Thyroid disease	9 (8%)	2 (10%)	7 (7%)	0.438
Main echocardiographic findings				
LA systolic diameter (PLAX), mm	40.4 ± 6.2	39.5 ± 3.8	40.5 ± 6.6	0.567
LA volume index, mL/m^2^	22.6 ± 13.1	26.1 ± 8.7	22.1 ± 13.6	0.335
RA volume index, mL/m^2^	8.7 ± 10.4	6.2 ± 8.3	9.7 ± 10.1	0.270
IV septum thickness, cm	1.18 ± 0.20	1.20 ± 0.15	1.19 ± 0.21	0.833
LV end-diastolic diameter index, cm/m^2^	2.55 ± 0.42	2.41 ± 0.22	2.58 ± 0.45	0.068
Posterior wall thickness, cm	1.00 ± 0.19	0.95 ± 0.15	1.00 ± 0.20	0.347
LV mass index, g/m^2^	107.5 ± 45.7	97.0 ± 48.8	110.4 ± 45.0	0.404
LV diastolic volume index. mL/m^2^	53.3 ± 12.3	50.2 ± 15.4	54.1 ± 11.6	0.513
LV ejection fraction	0.60 + 0.05	0.62 + 0.04	0.60 + 0.05	0.165
Laboratory samples				
Glycemia, mg/dL	96.4 ± 33.7	89.7 ± 29.4	96.4 ± 33.7	0.874
Creatinine, mg/dL	0.95 ± 0.22	0.98 ± 0.22	0.95 ± 0.22	0.918
Uric acid, mg/dL	4.9 ± 1.0	5.0 ± 1.0	4.9 ± 1.0	0.567
Total cholesterol, mg/dL	186.9 ± 37.6	183.8 ± 31.0	186.9 ± 37.6	0.322
LDL cholesterol, mg/dL	112.1 ± 37.8	115.1 ± 34.8	112.1 ± 37.8	0.754
Sodium, mEq/L	140.4 ± 3.3	141.3 ± 3.1	140.2 ± 3.3	0.281
Potassium, mEq/L	4.4 ± 0.5	4.5 ± 0.4	4.4 ± 0.5	0.372
Therapy				
Beta blockers	43 (36%)	12 (63%)	31 (31%)	0.016
Calcium antagonists	11 (9%)	3 (16%)	8 (8%)	0.530
Anti-platelet drugs	38 (32%)	8 (42%)	30 (30%)	0.447
Statins	56 (47%)	9 (47%)	47 (47%)	0.740
ACE-i or ARB	38 (32%)	7 (37%)	31 (31%)	0.806
Others	29 (24%)	6 (32%)	23 (23%)	0.585

Values are expressed mean ± SD for continuous variables and as number and percentage for categorical ones. Abbreviations. ACE-i = angiotensin-converting enzyme inhibitor; ARB = angiotensin receptor antagonist; IV = interventricular; LA = left atrium; LDL, low-density lipoprotein; LV = left ventricle; PLAX, parasternal long axis view; RA = right atrium; RV = right ventricle; and OSAS = obstructive sleep apnea syndrome.

**Table 2:** Sub-analysis of SAF patients.

	Class A and B (n = 10)	Class C (n = 9)	*p* Value
Age (years)	68 ± 11	74 ± 8	0.217
Males	6 (60%)	5 (56%)	0.605
Systolic blood pressure, mmHg	133 ± 17	121 ± 32	0.047
Diastolic blood pressure, mmHg	81 ± 9	99 ± 22	0.031
Resting heart rate, bpm	71 ± 4	80 ± 6	0.045
Body Mass Index, kg/m^2^	27.0 ± 3.2	25.3 ± 10.5	0.180
Body Surface Area, m^2^	1.9 ± 0.1	2.0 ± 0.2	0.344
Hypertension	9 (90%)	7 (78%)	0.458
Obesity	5 (50%)	5 (55%)	0.681
Chronic ischemic heart disease	2 (20%)	1 (11%)	0.542
Peripheral artery disease	1 (10%)	3 (33%)	0.249
History of cerebrovascular disease	3 (30%)	1 (11%)	0.333
History of palpitations/chest pain	9 (90%)	8 (89)	0.893
History of dyspnea on exercise	4 (40%)	3 (33%)	0.500
LV end-diastolic diameter, cm	4.8 ± 0.6	4.4 ± 0.5	0.471
Indexed LV end-diastolic diameter, cm/m^2^	2.4 ± 0.2	2.4 ± 0.2	0.797
IVS thickness, cm	0.12 ± 0.02	0.12 ± 0.01	0.462
LV ejection fraction	0.61 ± 0.5	0.63 ± 0.4	0.552
Indexed LA volume, mL/m^2^	24.6 ± 4.2	32.8 ± 4.6	0.022

Abbreviations as in Table 1.

## Data Availability

The data presented in this study are available on request from the corresponding author. The data underlying this article will be shared on reasonable request to the corresponding author.

## Supplementary Materials

The following supporting information can be downloaded at: https://www.mdpi.com/article/10.3390/jcdd10080336/s1, Figure S1: SAFARI study design; Table S1: Inclusion and exclusion criteria for patient enrolment.

## Appendix A

*SAFARI Steering Committee:* Cesare de Gregorio (Messina, Italy), Marco Di Franco, Salvatore Novo and Antonio V. Panno (Palermo, Italy), and Giuseppe Scaccianoce (Catania, Italy).

*SAFARI Study Group investigators* (Italy) and respective number of patients enrolled in the study: *City of Agrigento*: Riccardo Bentivegna (n = 1), Francesco Magro (n = 3), and Serena Magro (n = 5); *City of Caltanissetta*: Salvatore Pasqualetto (n = 2) and Luigi Scarnato (n = 4); *City of Catania*: Leonardo Costa (n = 4), Massimiliano Mulè (n = 2), Giuseppe Scaccianoce (n = 6), and Nidal Tourkmani (n = 2); *City of Messina*: Cesare de Gregorio (n = 8), Patrizia Grimaldi (n = 3), Santi Mangano (n = 4), Pietro Pugliatti (n = 6), and Antonino Recupero (n = 4); *City of Palermo*: Melania Bonocore (n = 2), Pino Caccamo (n = 2), Marcello Cavora (n = 2), Marco Di Franco (n = 8), Calogero Di Maio (n = 4), Giovanni Genco (n = 1), Antonino Giannola (n = 5), Vittorio Panno (n = 11), Rosa Rizzo (n = 8), and Maria Stellina Spoto (n = 2); Vincenzo Sucato (Core-lab); *City of Ragusa*: Angelo Paolo Dipasquale (n = 2), Vincenzo Manfrè (n = 4), Marco Miano (n = 4), and Salvatore Massimo Pètrina (n = 8); *City of Siracusa*: Francesco Coco (n = 2) and Salvatore Tummineri (n = 1); and *City of Trapani*: Giuseppe Brancatelli (n = 3) and Antonino Figlia (n = 2).
