# Supplementary material for: Subclinical Atrial Fibrillation on Prolonged ECG Holter Monitoring: Results from the Multicenter Real-World SAFARI (Silent Atrial Fibrillation ANCE-Sicily Research Initiative) Study
Source: J Cardiovasc Dev Dis. 2023 Aug 4;10(8):336. doi: 10.3390/jcdd10080336 (PMC10455667; doi:10.3390/jcdd10080336)
Supplement: Supplementary file 1 [file jcdd-10-00336-s001.zip › jcdd-2510776-supplementary.pdf]

Supplementary Materials

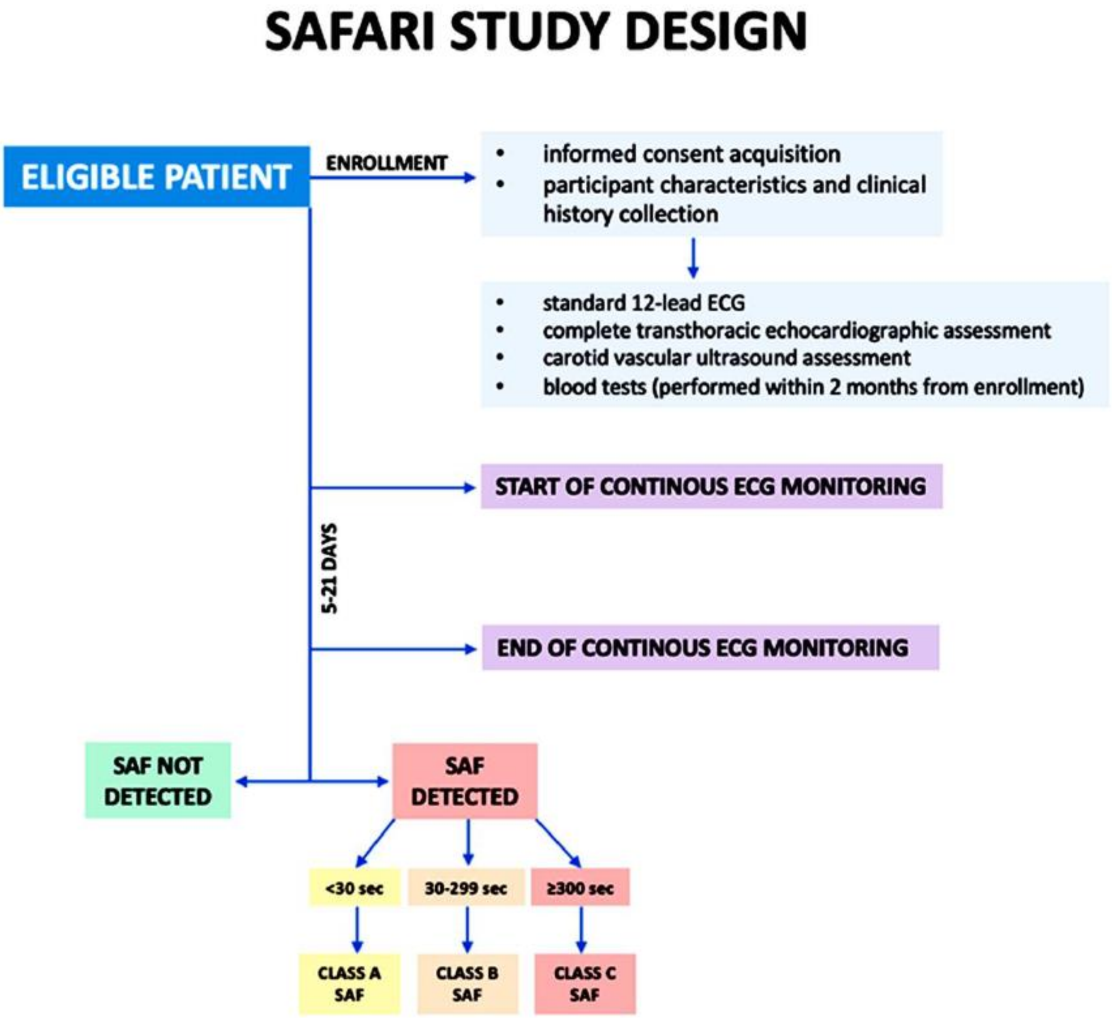

Figure S1. SAFARI study design.

Table S1. Inclusion and exclusion criteria for patient enrolment.

INCLUSION CRITERIA
Patients ≥55 years old, with at least one of the following conditions:
• Arterial hypertension
• Carotid and/or peripheral vascular disease (not critical)
• Chronic obstructive pulmonary disease (not on β2-agonists)
• Diabetes
• Dyslipidaemia
• Heart failure with preserved or mildly reduced ejection fraction (HFpEF or HFmrEF)
• Heart valve disease (no severe forms)
• Hypertrophic cardiomyopathy (no obstructive forms)
• Ischemic heart disease (stable)
• Obstructive sleep apnoea
• Overweight/obesity
• Pulmonary hypertension (pre-capillary)
• Renal disease (stages I-III)
• Smoking habit
• Thyroid disease (chronic)
and at least one of the following:
• History of palpitation
• Left atrial dilatation (>34 ml/m^2 or >22 cm^2)
• Right atrial dilatation (>30 ml/m^2 or >20 cm^2)
• Frequent premature atrial beats
• Previous transient ischemic attack (TIA)/minor stroke/gliosis
EXCLUSION CRITERIA
Any of the following:
• Age <55 years
• Cardiac amyloidosis or storage diseases
• Cardiac valve prosthesis
• Chronic hepatopathy or cirrhosis
• Chronic kidney disease (>III stage)
• First or second-degree atrioventricular block or sick sinus syndrome
• Heart failure with reduced ejection fraction (HFrEF)
• Malignancy
• Mitral valve prolapse with regurgitation grad 2-3 and/or mitral annular disjunction (MAD)
• History of atrial fibrillation
• Recent acute coronary syndrome
• Recent pulmonary edema (6 months)
• Severe aortic or mitral valve disease (stenosis/ regurgitation)
